# An Immunohistochemical Study of Cyclin D1 Expression in Astrocytic Tumors and its Correlation with Tumor Grade

**DOI:** 10.30699/ijp.2019.82024.1771

**Published:** 2019-08-01

**Authors:** Parvin Mahzouni, Fatemeh Taheri

**Affiliations:** 1 Department of Pathology, Isfahan University of Medical Sciences, Isfahan, Iran

**Keywords:** Glioblastoma, Immunohistochemistry, Cyclin D1, Neoplasm grading

## Abstract

**Background & Objective:**

Glioblastoma-multiforme is the high grade form of astrocytic tumors with a short survival time, which are the most common type of brain tumors. Therefore, finding new therapeutic options is essential. Cyclin D1 is expressed in some human malignancies and can be a potential target for therapeutic intervention. The aim of the present study was to determine this relationship.

**Methods:**

This is a cross-sectional study conducted in the pathology department of Al-Zahra Hospital in Isfahan, Iran. In this study, 100 samples diagnosed with astrocytic tumors between 2011 and 2015 that met the study’s requirements were studied and immunohistochemical staining for cyclin D1 was performed for each specimen. At the end, the relationship between the expression of cyclin D1 and various variables including tumor grades, tumor subtypes and patient demographic features were examined using appropriate statistical tests.

**Results:**

Of the 100 samples, cyclin D1 was positive in 60 samples and negative in 40 samples. Moreover, in 26 samples, the amount of the marker was low, while in 34 samples it was high. Following the results of the study, there was a significant difference (*P* =0.038) in the expression of the cyclin D1 marker among the four different grades of astrocytic tumors.

**Conclusion:**

The results showed that the expression of cyclin D1 was associated with different tumor grades, especially the high level of expression in grade 4, and the amount of cyclin D1 increased as the level of grade glioma increased.

## Introduction

Astrocytic tumors are the most common types of brain tumors. They originate in a particular type of glial cells (star-shaped brain cells located in the cerebrum) known as astrocytes. Glioblastoma multiforme is a highly aggressive astrocytic tumor, forming about 15.6% of all primary brain tumors, and is reported to be the most common form of malignant brain tumor (1). Glioblastoma is a rare disease with an incidence rate of 19.3 in every 100,000 people in the US, which is about 21 times less common than breast cancer (2, 3). This tumor may develop at any age, but its incidence rate is reported to be higher at 45-75 years of age (4). This tumor emerges more frequently as a primary tumor but is also likely to develop following low-grade astrocytoma (5).

Today, the standard treatment method for glioblastoma is surgical resection along with radiotherapy in combination with temozolomide. Considerable devel-opments in the treatment methods of glioblastoma has increased life expectancy among patients. This disease may last 14.6 months on average (6). Thus, an investigation into novel methods of treatment for the disease seems to be of significant importance. The cell cycle is controlled by three families of key regulator proteins, including cyclin, Cyclin-Dependent Kinase (CDK), and Cyclin-Dependent Kinase Inhibitor (CDKI). The kinds of CDK, cyclin and CDKI involved in the regulation of the cell cycle are different for each stage (7). Cyclin identifies specific kinases (CDKs), attaches itself to them, and forms complexes that allow for the development of the cell cycle. In the transition from G1 to S, first the cyclin D1-3 complex and then cyclin E are involved by activating CDK4-6 and CDK2 (8, 9). The activity of these complexes is controlled through phosphorylation and dephosphorylation mechanisms.

The cyclin D1 may modulate invasive ability by increasing the activity of matrix metalloproteinase (MMP) and enhancing cell movement (10). It has been demonstrated that the overexpression of cyclin D1 is correlated with higher tumor grade in papillary thyroid carcinoma (11), breast cancer (12), renal cell carcinoma (13) and prostate carcinoma (14). There is no comprehensive study on the evaluation of expression of cyclin in astrocytic tumors. Also, there seems to be a lack of agreement regarding the frequency of cyclin D1 in low-grade and high-grade astrocytic tumors. The present study was conducted to investigate the relationship between the grade of astrocytic tumors and the expression of cyclin D1. 

## Materials and Methods

This is a cross-sectional study conducted on the paraffin blocks available in the archives of Al-Zahra Hospital’s pathology lab from patients diagnosed with an astrocytic brain tumor between 2011 and 2015. The study was approved in the ethics committee of Isfahan University of Medical Sciences. (Approval code: Ir.mui.rec.1395.3.290)

All the samples collected from patients diagnosed with an astrocytic tumor between 2011 and 2015,were examined.

The samples of patients whose demographic and clinical characteristics were available and diagnosed with astrocystic tumors with paraffin blocks containing enough tissue for immunohistochemcal staining were studied. First, the H & E slides were re-examined to determine the various grades and subtypes of the tumors based on the latest tumor report classification system (Table 1).

**Table1:** WHO Classification of Tumors Affecting the Central Nervous System

**Grade**	**Subtype**
I	Subpendymal giant cell astrocytoma, pilocytic astrocytoma, , pleomorphic xanthoastrocytoma
II	Pilomyxmoid astrocytoma, protoplasmic astrocytoma , gemistocytic astrocytoma, fibrillary astrocytoma, mixed astrocytoma
III	Anaplastic astrocytoma
IV	Glioblastoma, giant cell glioblastoma, gliosarcoma

Then, 4 μm-thick sections were cut from the paraffin block and placed on poly-L-Lysine adhesive slides. Immunohistochemical staining was performed using the steptavidin-biotin-HRP complex method of Immunoperoxidase Secondary Detection System (Chemicon International, USA) according to the manufacturer’s protocols. Briefly, the sections were deparaffinized at 60°C for a minimum of 60 minutes, followed by incubation in xylene and hydration in a series of decreasing concentrations of ethanol of 100%, 70% and 30%. After that, the sections were placed in a microwavable plastic container and filled with antigen retrieval solution (0.01mol/L citrate buffer, PH 6.8). They were boiled in the microwave for 14 minutes for the purpose of antigen retrieval. The sections were washed in phosphate-buffered saline (PBS, pH 7.6), and immersed in 3% hydrogen peroxide and 100% methanol for 10 min to remove endogenous peroxidase activity. The primary antibody of cyclin D1, (Clone FP12) Rabbit Monoclonal Antibody (produced by DAKO in Denmark), was used in this study. The slides were incubated with primary antibodies at a dilution of 1:50 overnight at 4°C with cyclin D1 (clone FP12) Rabbit Monoclonal Antibody. After washing with PBS, the sections were incubated with biotinylated goat anti-mouse immunoglobulin (Dako Ltd) for 30 min at room temperature, washed with PBS, then incubated with streptavidin-peroxidase conjugate (1:500; Amersham Pharmacia Biotech, Bucks, U.K.) for 30 min. The sections were developed with diaminobenzidine tetrahydrochloride solution (Sigma-Aldrich, Poole, U.K) and 0.1% H2O2 and counterstained with haematoxylin for 30 seconds. The sections were dehydrated in ascending alcohols, then dried and mounted. At every stage of the staining process, positive and negative controls were set. Mantle cell lymphoma and follicular lymphoma were used to achieve positive and negative control, respectively.

Sections stained for cyclin D1 were examined by Olympus CX microscope (Malaysia) and the percentage of immunostained cells was determined. The percentages of cyclin D1 immunopositive tumor cells were counted in 5 consecutive microscopic fields (magnification 100x) per tumor sample in area, which showed the highest density of these cells. In each field, 100 tumor cell nuclei were evaluated and the mean for each of the 5 fields was calculated. A distinct granular brown nuclear stain was scored as positive. Only nuclear positivity, in at least 5% of tumor cell nuclei, was considered as positive. Samples from patients with <50% cyclin D1-positive tumor cells were considered low expressors, whereas those with >50% cyclin D1-positive tumor cells were considered high expressors (15).

Finally, the statistical analyses were carried out with SPSS (SPSS Inc., Chicago IL, USA) version 20, using the Chi-square, Mann Whitney and Independent t-test.

## Results

Among 100 samples, 10 samples (10%) were determined as grade I, 25 samples (25%) as grade II, 5 samples (5%) as grade III and 60 samples (60%) as grade IV.In the present study, the mean age of the enrolled patients was 46.53 ± 1.81 years. 59 patients (59%) were male and 41 (41%) were female

**Figure 1:**
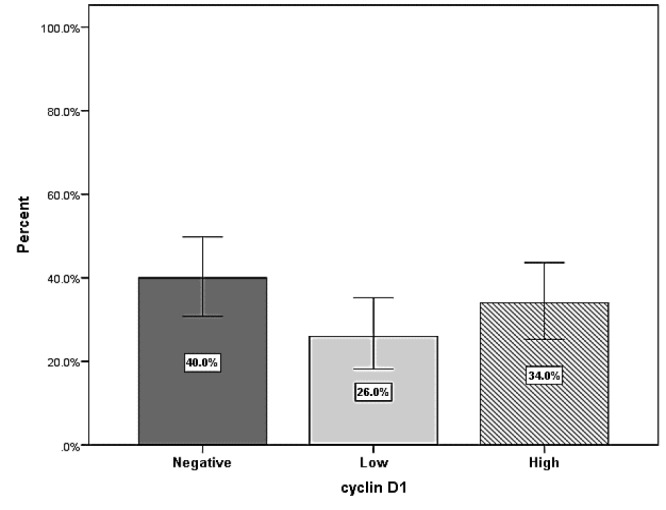
Expression level of cyclin D1 in astrocytic tumors

Cyclin D1 expression was positive in 60 samples (60%) and negative in 40 samples (40%). In 26 samples (26%) the expression level of cyclin D1 was low and in 34 samples (34%) highly expressed (Figure 1)..

The correlation between the expression of cyclin D1 with different grades of astrocytic tumors, is shown in table 2. Furthermore, it was also observed that the expression of cyclin D1 was significantly different (*P*= 0.038) among the four different grades of astrocytic tumors.

**Figure 2:**
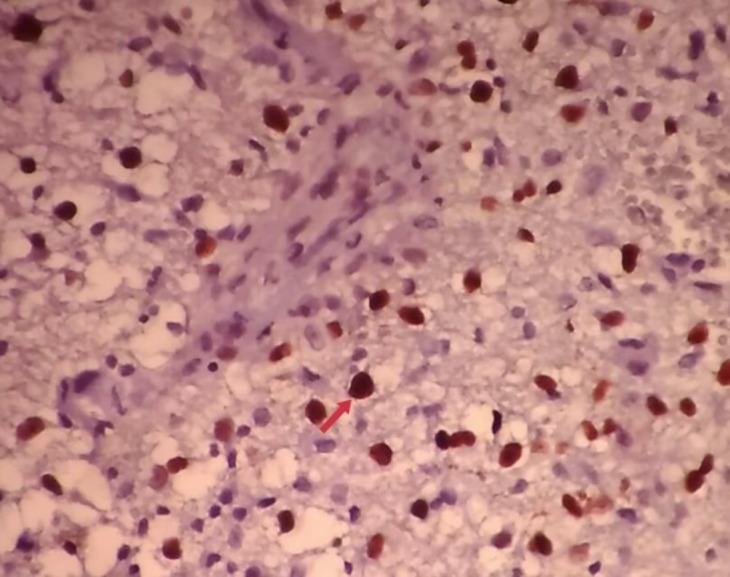
Glioblastoma multiforme grade IV, in a sample from the parietal lobe of the brain. The nuclei stained with cyclin D1 are marked with a red arrow (x 400)

Moreover, the expression of cyclin D1 was significantly different among different subtypes of astrocytic tumors (*P-* = 0.047). Of the 25 samples diagnosed with grade II tumors, one sample belonged to the Gemistocytic Astrocytoma subtype with a negative expression of cyclin D1 (table 3 and figure 2).

There was no correlation between the expression of cyclin D1 with the age and sex of patients. However, the average age of patients with high levels of cyclin D1 expression was higher than the other two groups.

**Table 2:** Expression level of cyclin D1 in different grades of astrocytic tumors

		**cyclin.D1**			**p-value**
		**Negative**	** Low**	**High**	
Grade	**grade one**	4	4	2	0.038	
		40.0%	40.0%	20.0%		
	**grade tow**	14	9	2		
		56.0%	36.0%	8.0%		
	**grade tree**	2	1	2		
		40.0%	20.0%	40.0%		
	**grade four**	20	12	28		
		33.3%	20.0%	46.7%		
Total		40	26	34		
		40.0%	26.0%	34.0%		

**Table 3:** Expression of cyclin D1 among different subtypes of astrocytic tumors

		**Cyclin D1**			**P-value**
		**Negative**	**Low**	**High**	
		4	4	2	
		40.0%	40.0%	20.0%	
	**Diffuse Fibrillary Astrocytoma**	13	9	2	0.047
		54.2%	37.5%	8.3%	
	**Anaplastic Astrocytoma**	2	1	2	
		40.0%	20.0%	40.0%	
	**Glioblastoma Multiforme**	20	12	28	
Total		33.3%	20.0%	46.7%	
		39	26	34	
		39.4%	26.3%	34.3%	

## Discussion

A large amount of supporting evidence suggests that changes in the cell cycle can play a crucial role in the pathogenesis of glioma (16). Numerous studies have demonstrated that cyclin D1 is closely related to cancers. An overexpression of cyclin D1 has been frequently associated with high-grade tumors such as breast tumors, colorectal tumors, prostate cancer, melanomas and lymphomas (17). The present study demonstrated that cyclin D1 is expressed in 60% of astrocytic tumors. A study conducted in 2007 reported that the expression of cyclin D1 protein was 40%, 12%, 52% and 58% in breast cancer, colon tumors, glioblastomas and melanomas (18). In a similar study conducted by Qu Dw et al. on gliomas in 2014, the expression of cyclin D1 in gliomas was 59% (19).

According to the results obtained from this study it was found that the expression of cyclin D1 was significantly different among four different grades of astrocytic tumors. In a study performed by Tan et al. in 2004 on 84 samples of glioma using immunohistochemistry, it was demonstrated that there is a significant difference in the expression of cyclin D1 between high-grade glioma and low-grade glioma. In this study, the expression of cyclin D1 in low-grade and high-grade gliomas was positive in 31.25% and 61.53% of samples, respectively (20). In addition, a study conducted by Zhang et al. in 2005 on 52 samples of gliomas, it was revealed that the expression of cyclin D1 is closely related to tumor formation and the progression of gliomas (21). 

In a similar study conducted by Qu Dw et al on gliomas in 2014, the expressions of cyclin D1 in normal brain tissue, low-grade gliomas and high-grade gliomas were reported to be 22%, 46% and 75%, respectively. However, in this study, the expression of Ki67 also showed a significant difference between normal brain tissue and high-grade gliomas (19). In another study by A Chakrabarty et al. on gliomas, similar results have been reported (22).

In this study, it has been shown that there is no significant relationship between the expression of cyclin D1 and the gender or age of the patient. A study conducted by Zhang yy et al. revealed that the overexpression of cyclin D1 can be related to advanced squamous cell carcinoma of larynx cancer, but it does not seem to be influenced by sex and age (23).

Although it has been reported that elevated cyclin D1 expression in human glioblastoma correlates with a poor prognosis (24, 25), one study demonstrated that higher cyclin D1 levels were present in the glioblastoma group (26). 

Generally, the results obtained from this study demonstrated that the expression level of cyclin D1 seems to be directly related to tumor grade, while it does not seem to be related to the sex and age of the patient. This study revealed that as the grade of astrocytic tumors increases, the expression level of cyclin D1 increases as well, suggesting that cyclin D1 can promote the development of glioma and, therefore, can be used as an indicator in the judgment of the prognosis of glioma. A study by Ling et al. demonstrated that reducing cyclin D1 protein levels can effectively aid in treating tumors induced by Notch-1, showing that cyclin D1 is an important protein functioning in tumor formation (27). Therefore, cyclin D1 may become a new target for the clinical treatment of cancer.

## Conclusion

In this study the high expression of cyclin D1 was observed in high-grade astrocytic tumors, particularly Glioblastoma multiforme. It was found that cyclin D1 could possibly be used as a target in the treatment of tumors.

## References

[1] Morana G, Tortora D, Stagliano S, Nozza P, Mascelli S, Severino M (2018). Pediatric astrocytic tumor grading: comparison between arterial spin labeling and dynamic susceptibility contrast MRI perfusion. Neuroradiology.

[2] Ostrom QT, Gittleman H, Farah P, Ondracek A, Chen Y, Wolinsky Y (2013). CBTRUS statistical report: Primary brain and central nervous system tumors diagnosed in the United States in 2006-2010. Neuro Oncol.

[3] Ferlay J, Soerjomataram I, Dikshit R, Eser S, Mathers C, Rebelo M (2015). Cancer incidence and mortality worldwide: sources, methods and major patterns in GLOBOCAN 2012. Int J Cancer.

[4] Raverot G, Wierinckx A, Dantony E, Auger C, Chapas G, Villeneuve L (2010). Prognostic factors in prolactin pituitary tumors: clinical, histological, and molecular data from a series of 94 patients with a long postoperative follow-up. J Clin Endocrinol Metab.

[5] Cai X, Sughrue ME (2018). Glioblastoma: new therapeutic strategies to address cellular and genomic complexity. Oncotarget.

[6] Gately L, McLachlan SA, Dowling A, Philip J (2017). Life beyond a diagnosis of glioblastoma: a systematic review of the literature. J Cancer Surviv.

[7] Koontongkaew S (2013). The tumor microenvironment contribution to development, growth, invasion and metastasis of head and neck squamous cell carcinomas. J Cancer.

[8] Liu S, Yin F, Fan W, Wang S, Guo XR, Zhang JN (2012). Over-expression of BMPR-IB reduces the malignancy of glioblastoma cells by upregulation of p21 and p27Kip1. J Exp Clin Cancer Res.

[9] Zagzag D, Blanco C, Friedlander DR, Miller DC, Newcomb EW (2003 ). Expression of p27KIP1 in human gliomas: relationship between tumor grade, proliferation index, and patient survival. Hum Pathol.

[10] Arato-Ohshima T, Sawa H (1999). Over-expression of cyclin D1 induces glioma invasion by increasing matrix metalloproteinase activity and cell motility. Int J Cancer.

[11] Lee SH, Lee JK, Jin SM, Lee KC, Sohn JH, Chae SW (2010). Expression of cell-cycle regulators (cyclin D1, cyclin E, p27kip1, p57kip2) in papillary thyroid carcinoma. Otolaryngol Head Neck Surg.

[12] Aaltonen K, Amini RM, Landberg G, Eerola H, Aittomaki K, Heikkila P (2009). Cyclin D1 expression is associated with poor prognostic features in estrogen receptor positive breast cancer. Breast Cancer Res Treat.

[13] Lima MS, Pereira RA, Costa RS, Tucci S, Dantas M, Muglia VF (2014). The prognostic value of cyclin D1 in renal cell carcinoma. Int Urol Nephrol.

[14] Pereira RA, Ravinal RC, Costa RS, Lima MS, Tucci S, Muglia VF (2014). Cyclin D1 expression in prostate carcinoma. Braz J Med Biol Res.

[15] Nan KJ, Jing Z, Gong L (2004). Expression and altered subcellular localization of the cyclin-dependent kinase inhibitor p27Kip1 in hepatocellular carcinoma. World J Gastroenterol.

[16] Wu WS, Chien CC, Liu KH, Chen YC, Chiu WT (2017). Evodiamine Prevents Glioma Growth, Induces Glioblastoma Cell Apoptosis and Cell Cycle Arrest through JNK Activation. Am J Chin Med.

[17] Fu M, Wang C, Li Z, Sakamaki T, Pestell RG (2004). Minireview: Cyclin D1: normal and abnormal functions. Endocrinology.

[18] Kleiner HE, Krishnan P, Tubbs J, Smith M, Meschonat C, Shi R Tissue microarray analysis of eIF4E and its downstream effector proteins in human breast cancer J Exp Clin Cancer Res. 2009.

[19] Qu DW, Xu HS, Han XJ, Wang YL, Ouyang CJ (2014). Expression of cyclinD1 and Ki-67 proteins in gliomas and its clinical significance. Eur Rev Med Pharmacol Sci.

[20] Tan PG, Xing Z, Li ZQ (2004). [Expression of cyclin D1 in brain gliomas and its significance]. Ai Zheng.

[21] Zhang X, Zhao M, Huang AY, Fei Z, Zhang W, Wang XL (2005). The effect of cyclin D expression on cell proliferation in human gliomas. J Clin Neurosci.

[22] Chakrabarty A, Bridges LR, Gray S (1996). Cyclin D1 in astrocytic tumours: an immunohistochemical study. Neuropathol Appl Neurobiol.

[23] Kouraklis G, Theocharis S, Vamvakas P, Vagianos C, Glinavou A, Giaginis C (2006). Cyclin D1 and Rb protein expression and their correlation with prognosis in patients with colon cancer. World J Surg Oncol.

[24] Wang J, Wang Q, Cui Y, Liu ZY, Zhao W, Wang CL (2012). Knockdown of cyclin D1 inhibits proliferation, induces apoptosis, and attenuates the invasive capacity of human glioblastoma cells. J Neurooncol.

[25] Li M, Xiao A, Floyd D, Olmez I, Lee J, Godlewski J (2017). CDK4/6 inhibition is more active against the glioblastoma proneural subtype. Oncotarget.

[26] Camacho-Urkaray E, Santos-Juanes J, Gutierrez-Corres FB, Garcia B, Quiros LM (2018). Establishing cut-off points with clinical relevance for bcl-2, cyclin D1, p16, p21, p27, p53, Sox11 and WT1 expression in glioblastoma - a short report. Cell Oncol.

[27] Ling H, Jolicoeur P (2013). Notch-1 signaling promotes the cyclinD1-dependent generation of mammary tumor-initiating cells that can revert to bi-potential progenitors from which they arise. Oncogene.

